# Long noncoding RNA and messenger RNA profiling in epicardial adipose tissue of patients with new-onset postoperative atrial fibrillation after coronary artery bypass grafting

**DOI:** 10.1186/s40001-024-01721-x

**Published:** 2024-02-17

**Authors:** Yuanshu Peng, Pixiong Su, Lei Zhao

**Affiliations:** Heart Center & Beijing Key Laboratory of Hypertension, Beijing Chaoyang Hospital, Capital Medical University, Beijing, 100020 China

## Abstract

**Background:**

Postoperative atrial fibrillation (POAF) constitutes a significant complication following coronary artery bypass graft surgery (CABG), potentially linked to epicardial adipose tissue (EAT). This investigation seeks to elucidate the association between POAF and EAT at the genetic level.

**Methods:**

EAT and clinical data from patients undergoing CABG were systematically acquired, adhering to established inclusion and exclusion criteria. Patients were categorized into POAF and Non-POAF groups based on the presence or absence of POAF. High-throughput sequencing data of EAT were subjected to differential expression analysis and gene function assessment. A random selection of long noncoding RNAs (lncRNAs) underwent quantitative real-time polymerase chain reaction (qRT-PCR) for validation of the high-throughput sequencing findings. Coexpression analysis was employed to elucidate the interactions between lncRNAs and messenger RNAs (mRNAs).

**Results:**

RNA sequencing yielded a total of 69,685 transcripts (37,740 coding and 31,945 noncoding sequences), representing 16,920 genes. Within this dataset, 38 mRNAs and 12 lncRNAs exhibited differential expression between the POAF and Non-POAF groups (*P* < 0.05, fold change > 1.5). The qRT-PCR results for lncRNAs corroborated the sequencing findings (*P* < 0.01). Functional enrichment analysis of genes and the coexpression network indicated that these differentially expressed RNAs were primarily implicated in processes such as cell growth, differentiation, signal transduction, as well as influencing tissue fibrosis and ion transmembrane transport.

**Conclusions:**

This study unveils a potential association between myocardial fibrosis and ion channels co-regulated by mRNAs and lncRNAs, closely linked to the emergence of new-onset POAF, after accounting for clinical risk factors. This discovery holds promise for further advances in clinical and fundamental research.

**Supplementary Information:**

The online version contains supplementary material available at 10.1186/s40001-024-01721-x.

## Highlights

1. This study describes, for the first time, the RNA expression profile of EAT in patients with non-valvular POAF
2. We identified that the LncRNA expression profile of EAT was closely related to the occurrence of PAOF
3. We found that the co-regulation of mRNAs and LncRNAs on myocardial fibrosis or ion channel may be closely related to the occurrence.

**Supplementary Information:**

The online version contains supplementary material available at 10.1186/s40001-024-01721-x.

## Introduction

There has been a noteworthy rise in the number of hospitalized patients globally, suffering from atrial fibrillation, in recent years. This surge has been linked to an increased incidence of ischemic stroke, heart failure, and notably elevated mortality rates. Consequently, it has placed a substantial burden on both patients and the healthcare system [1]. Among the most prevalent complications is the emergence of new-onset atrial fibrillation following coronary artery bypass grafting (CABG), occurring in approximately 10–35% of cases [2–6]. New-onset postoperative atrial fibrillation (New-onset POAF) is associated with a heightened risk of all-cause mortality within 30 days post-surgery, as well as an increased likelihood of complications such as stroke, cardiac insufficiency, renal insufficiency, prolonged hospitalization, extended intensive care unit monitoring, and prolonged ventilator use. Beyond its impact on postoperative recovery, POAF places a significant financial strain on patients, their families, and society at large. Long-term studies reveal a 5 year all-cause mortality rate of 16.3% in CABG patients with atrial fibrillation, a figure significantly higher than that of non-atrial fibrillation patients [13].

The onset of atrial fibrillation is influenced by various factors, including advanced age, atrial fibrosis, electrolyte imbalances, and other variables. Research findings [7] indicate that fluctuations in autonomic nervous system activity may represent a significant mechanism contributing to the heightened susceptibility to paroxysmal atrial fibrillation in diabetic patients. A recurring challenge in the treatment of atrial fibrillation is the reoccurrence of the condition after ablation procedures. Sardu et al. [8] observed an association between elevated levels of sarcoplasmic reticulum calcium ATPase (SERCA) and a reduced risk of post-ablation atrial fibrillation recurrence. Similarly, miRNA [9] and the overexpression of inflammatory pathways [10] have been linked to the recurrence of atrial fibrillation after radiofrequency catheter ablation. These factors can be utilized to evaluate patients' responsiveness to radiofrequency catheter ablation treatment. However, Sardu et al. [10] have affirmed that anti-inflammatory therapy has not yielded success in preventing atrial fibrillation recurrence after ablation treatment.

Adipose tissue serves as a vital endocrine organ within the human body [11]. Epicardial adipose tissue (EAT) is situated on the surface of the atrium and ventricle, enclosed by the pericardium, with a prominent presence on the surface of the right atrium and anterior interventricular sulcus [12]. It resides in close proximity to cardiomyocytes and can secrete various proinflammatory molecules that exert a localized influence on myocardial cells [13]. Inflammation of EAT plays a pivotal role in adverse cardiovascular outcomes following coronary artery bypass grafting [14]. Recent research [15] has revealed that the secretion of adipo-fibrokines, such as activin A, can promote myocardial fibrosis, subsequently contributing to the development of atrial fibrillation. Furthermore, studies have confirmed the intrinsic correlation between the gene expression profile of EAT (both protein-coding and noncoding genes) and the occurrence of atrial fibrillation [16]. Risk factors associated with atrial fibrillation may activate specific genes, thereby participating in the pathological processes that lead to atrial fibrillation [17].

Nonetheless, the mechanisms underlying POAF, particularly the genetic factors linked to epicardial adipose gene expression profiles, remain unclear.

We posit that the RNA expression profile in EAT may bear a close association with the emergence of POAF. The objective of this study was to investigate the pivotal genes potentially implicated in the onset and progression of POAF following CABG through RNA sequencing (RNA-seq) of EAT. In addition, we aimed to predict their functions and involvement in regulatory pathways using bioinformatics analysis. These efforts seek to unveil the plausible mechanisms behind POAF, laying a theoretical foundation for the prevention and treatment of POAF post-CABG.

## Materials and methods

### Study population and sample

Patients who underwent coronary artery bypass surgery at the Department of Cardiac Surgery, Beijing Chaoyang Hospital, Capital Medical University, during the period from March 2021 to August 2021, were enrolled according to specified inclusion and exclusion criteria. Individuals meeting any of the exclusion criteria were excluded from the study. Inclusion criteria encompassed patients who were (1) 18 years of age or older, (2) subjected to coronary artery bypass surgery, and (3) devoid of a prior history of arrhythmia. Exclusion criteria encompassed patients who underwent (1) emergency surgery, (2) repeated bypass surgery, (3) concurrent valve surgery or other cardiac procedures, and (4) employment of cardiopulmonary bypass.

Our previous study reported a POAF incidence ranging from 11 to 16% within our institution. For the present investigation, we planned to conduct RNA-seq analysis on 10 specimens (5 in the POAF group and 5 in the Non-POAF group); therefore, a total of 50 EAT specimens were procured for this purpose.

The classical off-pump CABG technique was employed in patients with a median sternal incision, whereas patients with endoscope-assisted small incisions received left intercostal small incisions. The proximal end of the free bridge was anastomosed with the ascending aorta, and the "Heartstring non-blocking proximal anastomosis system" was consistently utilized. During the operation, ultrasonic instantaneous blood flow measurement techniques were employed to assess the blood flow in the bridging vessels.

EAT samples were collected from the surface of the right atrium, in close proximity to the superior vena cava, using a conventional scalpel (not an electric knife). This occurred after the pericardium was opened but prior to the initiation of the bypass procedure. Subsequently, these samples were promptly snap-frozen in liquid nitrogen and preserved at − 80 °C until ribonucleic acid extraction. The study protocol obtained approval from the Beijing Chaoyang Hospital Ethics Committee and adhered to the principles outlined in the Declaration of Helsinki.

### Definition and detection of new-onset POAF

Continuous electrocardiogram (ECG) monitoring was carried out for 24 h in the ICU post-surgery. Once the patient was transferred to the general ward, a 12-lead ECG was promptly conducted to investigate any associated symptoms. Atrial fibrillation often manifested at various intervals during the ICU stay, with ECG revealing atrial fibrillation episodes lasting more than 30 s. In the general ward, the occurrence of atrial fibrillation was assessed based on related symptoms and the results of the 12-lead ECG. It is important to note that spontaneous conversion from atrial fibrillation to sinus rhythm within 24 h was not observed in this study, necessitating medical cardioversion.

### lncRNA and mRNA expression profiling by RNA-seq

Total RNA, inclusive of long noncoding RNAs (lncRNAs), was extracted from freshly frozen EAT employing the TrIzol reagent (Invitrogen, CA, USA). Approximately 1–2 µg of total RNA from each sample underwent quantification using a NanoDrop ND-1000 instrument and qualification through agarose gel electrophoresis. RNA isolation from the total RNA (ribosomal RNA removed) was performed using an NEBNext Poly(A) mRNA Magnetic Isolation Module (New England Biolabs). This RNA was then utilized for RNA-seq library preparation, employing a KAPA Stranded RNA-Seq Library Prep Kit (Illumina). To assess the libraries, their concentration, fragment size distribution (ranging between 400 and 600 bp), and adapter dimer contamination were evaluated using an Agilent 2100 Bioanalyzer. Sequencing was conducted using the Illumina HiSeq 6000 (Illumina, CA, USA), following the manufacturer's protocols, to yield raw sequencing data for quality control purposes.

The NanoDrop ND-1000 instrument was utilized to gauge the concentration (abs 260) and assess protein contamination (ratio abs260/abs230) within the total RNA samples. Subsequent to quality control, fragments underwent trimming through the 5′ and 3′ adapters, with reads of ≤ 20 bp being filtered out using Cutadapt software. Ultimately, the processed, clean reads were mapped to the reference genome using Hisat 2 software to identify expression profiles. Scatter and volcano plots were generated to visualize the differentially expressed genes within the statistical computing and graphics environments of R, Python, or shell.

### Differential expression analysis of mRNAs and lncRNAs

The expression levels, quantified as fragments per kilobase of transcript per million mapped reads (FPKM values), were computed for known genes and transcripts using the R package ballgown. These FPKM values were derived from transcript abundances estimated by StringTie. The number of identified genes and transcripts per group was determined based on a mean FPKM in the group of ≥ 0.5. Subsequent differential expression analyses for genes and transcripts were conducted employing the R package ballgown, with criteria for filtering including a fold change (cutoff 1.5), *P* value (≤ 0.05), and FPKM (≥ 0.5 in one group).

### qRT-PCR validation of lncRNAs

RNA obtained through the TrIzol (Invitrogen) method underwent reverse transcription to produce cDNA using SuperScript III Reverse Transcriptase (Invitrogen) with the Gene Amp PCR System 9700 (Applied Biosystems). Real-time quantitative reverse transcriptase–polymerase chain reaction (qRT-PCR) was executed using a QuantStudio 5 Real-time PCR System (Applied Biosystems). The housekeeper gene β-actin, whose expression remained relatively constant across different samples, served as the internal reference to correct for quantitative RNA concentration variations and reverse transcription efficiency errors. This allowed for the determination of relative expression levels using the 2^−ΔΔCT^ method. Detailed primer information can be found in Additional file 1: Table S1.

### Gene Ontology and pathway analysis

The GO initiative (http://www.geneontology.org) defines gene attributes across three structured categories: biological process (BP), molecular function (MF), and cellular component (CC). Pathway analysis was conducted on the differentially expressed genes based on the latest Kyoto Encyclopedia of Genes and Genomes (KEGG) database (http://www.genome.jp/kegg). It aimed to identify whether the differentially expressed mRNAs were enriched in specific biological pathways. A Fisher’s exact test was employed to evaluate the statistical significance of such enrichments between the two groups, with a *P* value < 0.05 indicating statistical significance.

### Construction of a coexpression network

The combined analysis of lncRNA and mRNA was conducted to infer the function of lncRNA. The correlation coefficient between lncRNA and mRNA was determined based on the following criteria: Pearson’s correlation coefficient ≥ 0.8 and a *P* value < 0.05. Cytoscape (version 3.9.1) was employed to construct a coexpression network between lncRNA and mRNA.

### Statistical analysis

Statistical analyses were performed using SPSS Statistics 26.0 (IBM). Continuous measurement data were presented as mean ± standard deviation (*x* ± *s*). Hypothesis testing was conducted using the two-independent-samples *t* test. Categorical data were expressed as percentages, and differences between groups for unordered binary and multicategorical variables were assessed using the *χ*^2^ test or Fisher’s exact probability method.

## Results

### Baseline clinical characteristics of the study population

Out of the 50 EAT samples, six were from patients with POAF. One sample did not meet the criteria for RNA quantification and quality assurance, while the remaining five samples were valid. In the Non-POAF group, 44 patients were included after 1:1 propensity score matching. The primary clinical characteristics are summarized in Table 1. Perioperative basic clinical data showed no statistically significant differences in age, prevalence of hypertension, or diabetes between the two groups. There were also no statistically significant differences in postoperative drainage volume and the number of anastomoses. However, the levels of total cholesterol (TC) (2.8 ± 1.0 vs 4.6 ± 0.5, *P* = 0.005) and triglycerides (TG) (1.0 ± 0.5 vs 2.3 ± 0.8, *P* = 0.013) were significantly lower in the POAF group compared to the Non-POAF group. The low-density lipoprotein (LDL) levels exhibited a similar trend but did not reach statistical significance (1.6 ± 0.8 vs 2.6 ± 0.8, *P* = 0.100).

**Table 1:** Demographic and clinical characteristics of patient

Characteristics	POAF(*n* = 5)	Non-AF(*n* = 5)	*P* value
Age, years	70.8 ± 4.6	69.0 ± 6.0	0.609
Male, *n*,%	3 (60.0%)	3 (60.0%)	1.000
BMI, Kg/m^2^	24.4 ± 1.5	25.8 ± 1.4	0.168
Diabetes, *n*,%	2 (40.0%)	3 (60.0%)	1.000
Hypertension, *n*,%	3 (60.0%)	4 (80.0%)	1.000
Leukocytes, × 10^9^/L	6.0 ± 1.3	6.3 ± 1.3	0.710
Neutrophils	3.5 ± 0.8	3.6 ± 1.0	0.822
Lymphocytes	1.7 ± 0.6	2.0 ± 0.5	0.340
Neutrophil ratio	56.1 (54.4–62.1)	56.1 (53.8–58.2)	0.690
Lymphocytes ratio	27.9 (26.0–32.9)	34.1 (32.8–35.9)	0.421
Hemoglobin, g/L	136.6 ± 23	135.2 ± 22.2	0.925
BNP, ng/L	103.8 ± 75.5	79.0 ± 58.7	0.578
GLU,	6.7 ± 3.5	7.6 ± 2.8	0.666
HbA1C	6.1 ± 0.6	6.9 ± 1.4	0.083
TC, mmol/L	2.8 ± 1.0	4.6 ± 0.5	0.005^*^
LDL, mmol/L	1.6 ± 0.8	2.6 ± 0.8	0.100
HDL, mmol/L	0.76 ± 0.2	0.82 ± 0.1	0.584
TG, mmol/L	1.0 ± 0.5	2.3 ± 0.8	0.013^*^
Lpa, mmol/L	19.2 ± 12.7	14.9 ± 7.4	0.536
BUN, mmol/L	5.5 ± 1.4	6.9 ± 1.4	0.171
Scr, μmol/L	60.8 ± 13.1	77.0 ± 18.9	0.153
LVEDD, mm	50 ± 2	45.8 ± 2.5	0.019
LVEF, %	53.6 ± 10.4	62.8 ± 2.2	0.056
LAD, mm	41.9 ± 3.7	39.9 ± 1.9	0.316
RAD, mm	40.3 ± 3.4	38.8 ± 2.0	0.425
Anastomoses	2.6 ± 1.5	2.2 ± 1.1	0.645
Drainage-1	362.0 ± 88.1	388 ± 65.7	0.611
Drainage-T	977.0 ± 558.3	958.0 ± 306.5	0.948
Length of ICU stay	5.4 ± 1.5	4.6 ± 2.1	0.506

BMI: Body mass index; Drainage-1: BNP: Brain Natriuretic Peptide; HbA1C: Glycosylated Hemoglobin; TC: Total Cholesterol; LDL: Low Density Lipoprotein; HDL: High Density Lipoprotein; TG: Triglyceride; Lpa: Lipoprotein A; BUN: Blood Urea Nitrogen; Scr: Serum creatinine; LVEDD: left ventricular end-diastolic dimension; LVEF: left ventricular ejection fraction; LAD: Left atrial diameter; RAD: Right atrial diameter; Drainage-1: Drainage on the first postoperative day; Drainage-T: Total postoperative drainage; ICU: Intensive care unit

Medication upon admission and medication upon discharge are detailed in Additional file 4: Table S4. Subgroup analyses have been conducted separately for diabetic and non-diabetic patient cohorts, and the outcomes will be presented in Additional file 3: Table S3.

### lncRNA and mRNA sequencing results and differential expression

In EAT, RNA-seq yielded 69,685 transcripts, encompassing 37,740 coding sequences and 31,945 noncoding sequences, corresponding to 16,920 genes. This gene set consisted of 13,902 protein-coding genes and 3018 noncoding genes. Table 2 presents the top 30 transcripts, including both protein-coding and non-protein coding sequences. Some of these highly expressed transcripts are known to play pivotal roles in cardiovascular diseases, particularly in patients with type 2 diabetes mellitus. For example, MT-ATP6 is regulated by miR-378a and affects cardiac function by influencing ATP synthase function [18]. LncRNA RMBP has been attributed significant therapeutic value in the context of myocardial dysfunction [19].

**Table 2:** The top 30 expressed transcripts in epicardial adipose tissue

Gene ID	Gene name	Transcript type	Locus (chromosome)	Strand
ENSG00000283029	AL139099.5	non_coding	chr14	+
ENSG00000269900	RMRP	lincRNA	chr9	–
ENSG00000198899	MT-ATP6	protein_coding	chrM	+
ENSG00000228253	MT-ATP8	protein_coding	chrM	+
ENSG00000198886	MT-ND4	protein_coding	chrM	+
ENSG00000198888	MT-ND1	protein_coding	chrM	+
ENSG00000198804	MT-CO1	protein_coding	chrM	+
ENSG00000198763	MT-ND2	protein_coding	chrM	+
ENSG00000198938	MT-CO3	protein_coding	chrM	+
ENSG00000259001	AL355075.4	antisense_RNA	chr14	−
ENSG00000198727	MT-CYB	protein_coding	chrM	+
ENSG00000198712	MT-CO2	protein_coding	chrM	+
ENSG00000198786	MT-ND5	protein_coding	chrM	+
ENSG00000212907	MT-ND4L	protein_coding	chrM	+
ENSG00000251562	MALAT1	lincRNA	chr11	+
ENSG00000198840	MT-ND3	protein_coding	chrM	+
ENSG00000087086	FTL	protein_coding	chr19	+
ENSG00000156508	EEF1A1	protein_coding	chr6	−
ENSG00000276232	AC006064.5	sense_intronic	chr12	+
ENSG00000198695	MT-ND6	protein_coding	chrM	−
ENSG00000179914	ITLN1	protein_coding	chr1	−
ENSG00000170323	FABP4	protein_coding	chr8	−
ENSG00000123689	G0S2	protein_coding	chr1	+
ENSG00000205542	TMSB4X	protein_coding	chrX	+
ENSG00000167658	EEF2	protein_coding	chr19	−
ENSG00000034510	TMSB10	protein_coding	chr2	+
ENSG00000143196	DPT	protein_coding	chr1	−
ENSG00000197958	RPL12	protein_coding	chr9	−
ENSG00000004776	HSPB6	protein_coding	chr19	−
ENSG00000125730	C3	protein_coding	chr19	−

**Fig. 1:**
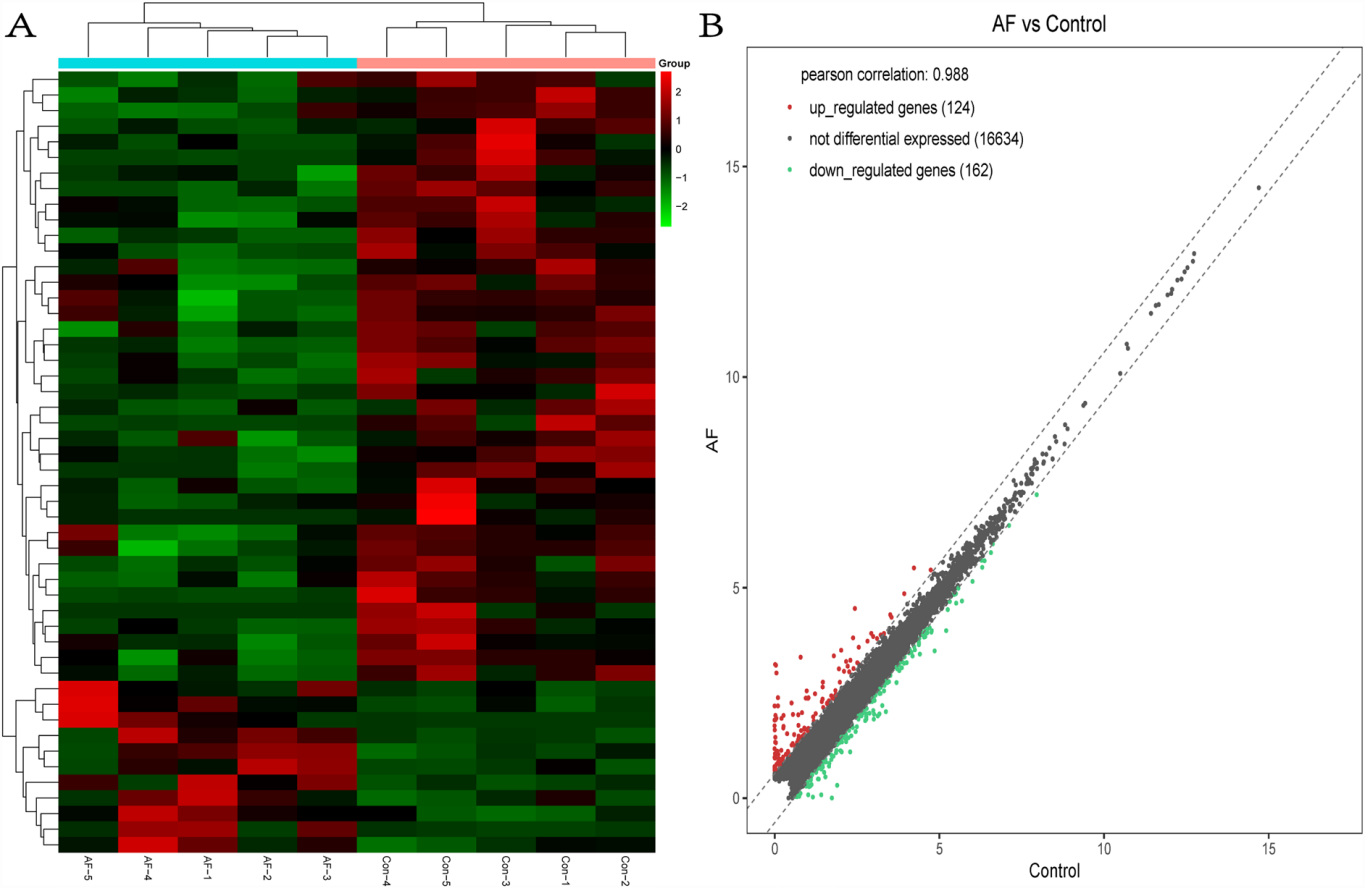
Clustering heatmaps (**A**) and scatterplots of differentially (**B**) expressed RNA-seq

**Fig. 2:**
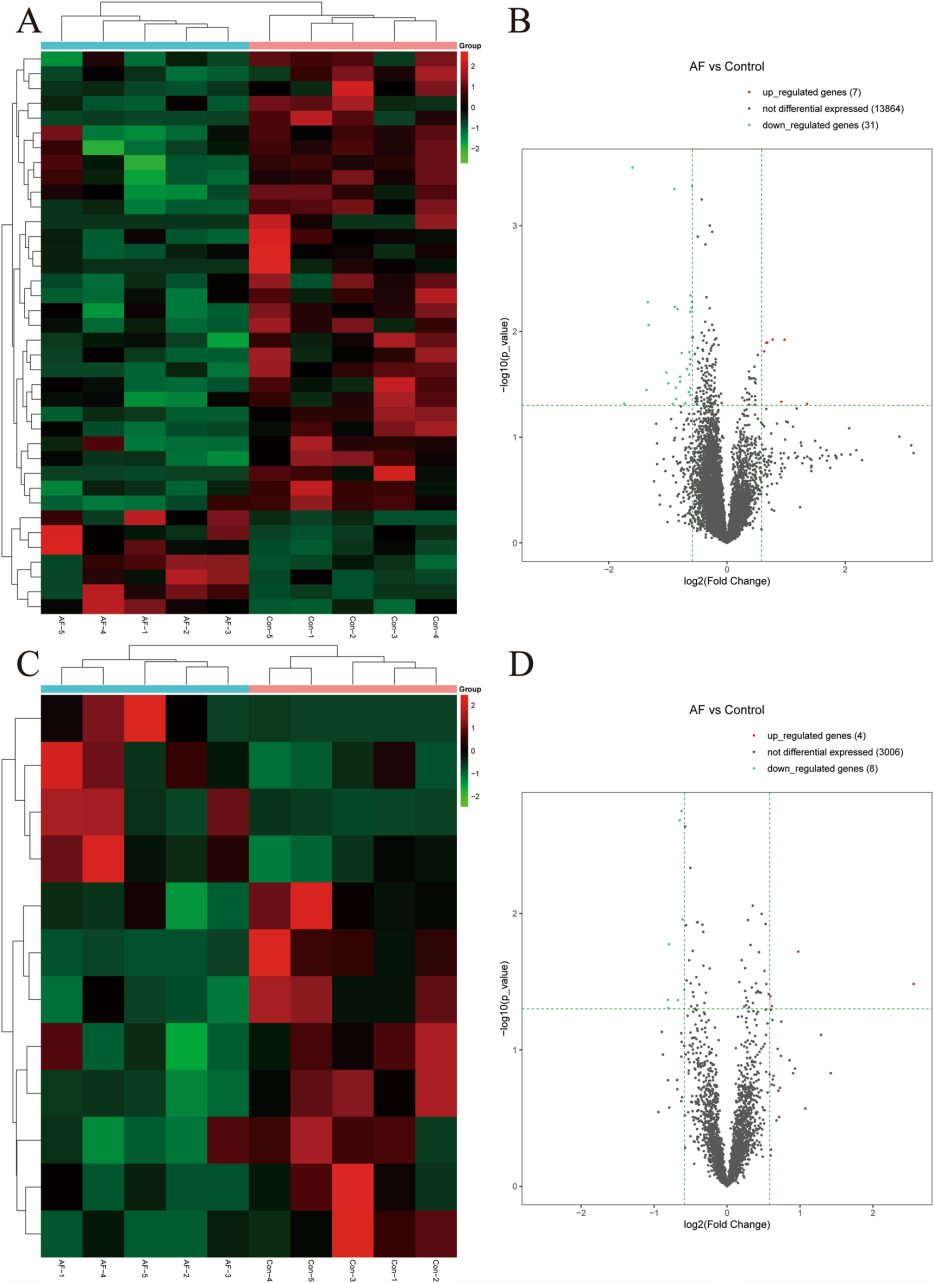
Clustering heatmaps and volcano plots of differentially expressed (*P* < 0.05, fold change > 1.5) mRNAs (**A**, **B**) and lncRNAs (**C**, **D**)

Differential expression analysis, depicted in Fig. 1, revealed 286 genes with differential expression between the POAF and Non-POAF groups. This included 11 significantly upregulated sequences and 39 significantly downregulated sequences (*P* < 0.05, |log2FC|≥ 0.585). Figure 1A illustrates a predominance of downregulated genes in the POAF group, while Fig. 1B underscores that despite the abundant RNA expression in EAT, the number of differentially expressed genes remained limited. In patients with POAF, gene expression of both long noncoding RNA (lncRNA) and messenger RNA (mRNA) in EAT exhibited significant differences from the Non-POAF group. Specifically, 7 mRNA expression levels were upregulated, and 31 were downregulated, along with 4 upregulated and 8 downregulated lncRNA expression levels (*P* < 0.05, |log2FC|≥ 0.585) (Fig. 2).

**Fig. 3:**
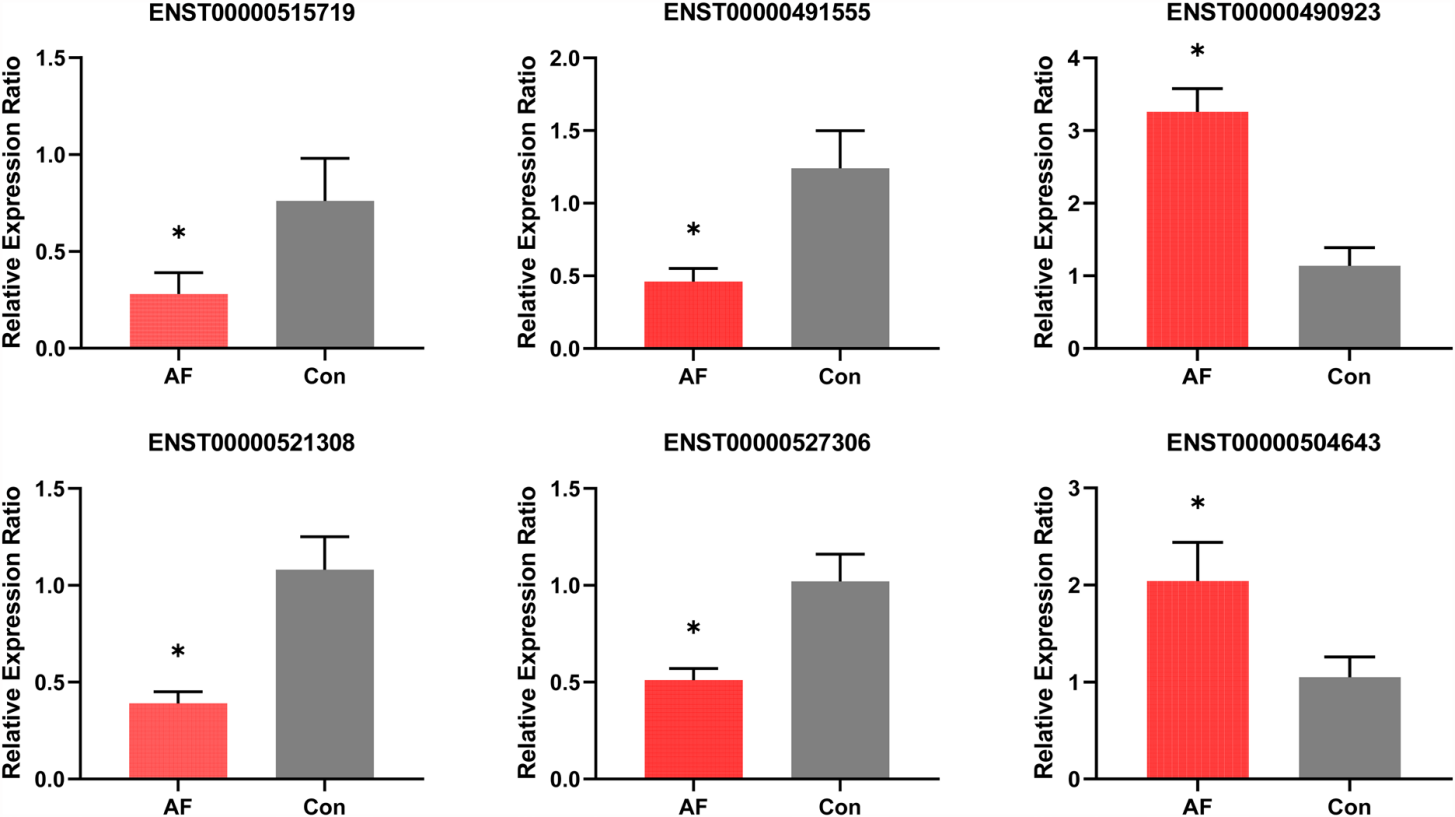
qRT-PCR validation results of randomly selected differentially expressed lncRNAs with *P* values less than 0.01

To validate the sequencing results, six randomly selected lncRNAs, chosen from the co-expression selected group (comprising 12 downregulated and 9 upregulated lncRNAs), underwent testing via qRT-PCR. The results demonstrated a consistent trend with the sequencing findings, with a significance level of *P* < 0.01 (Fig. 3).

### GO and KEGG pathway analysis of differentially expressed genes

**Fig. 4:**
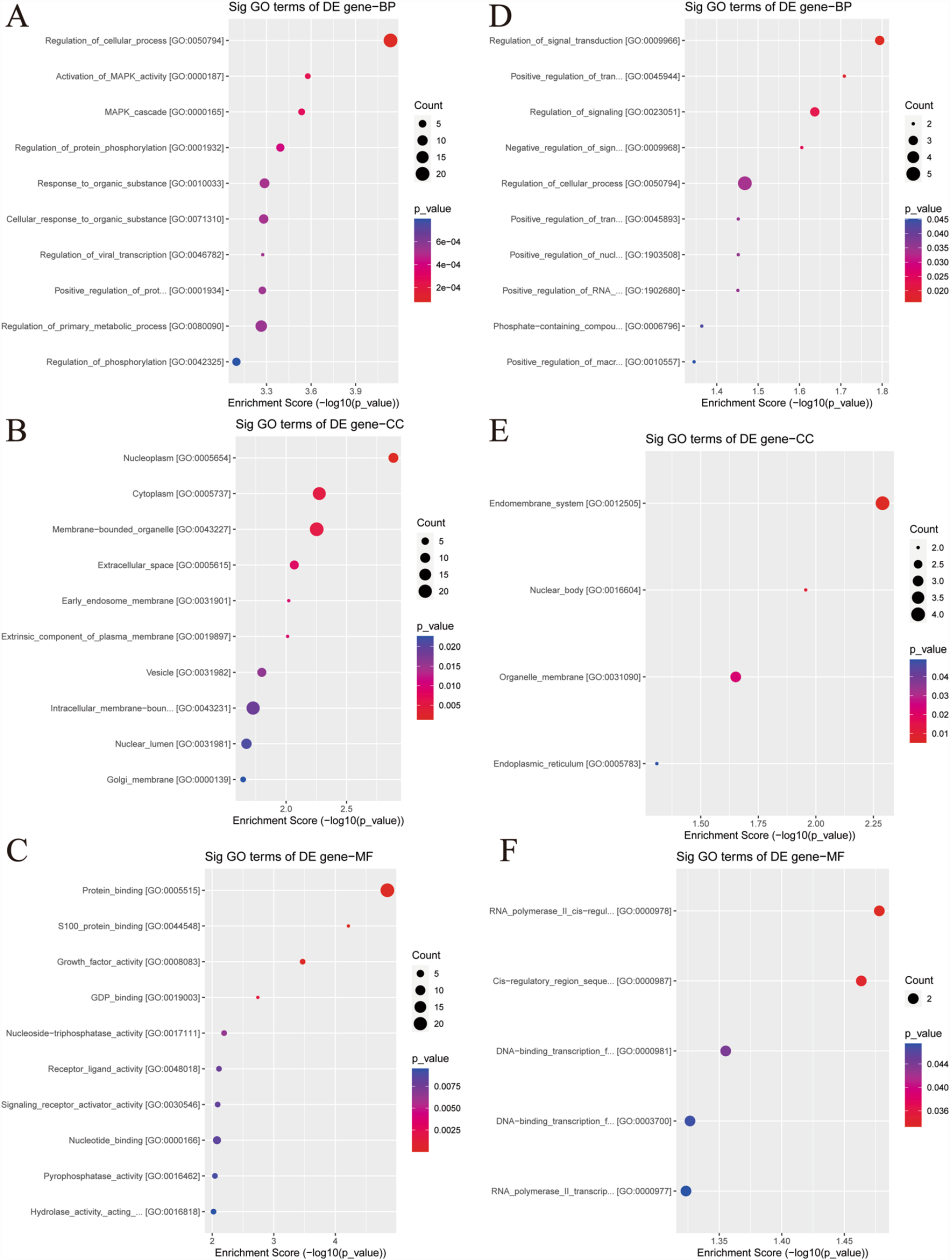
Top 10 significantly downregulated (**A**–**C**) and upregulated (**D**–**F**) items in descending order of enrichment score are presented as DotPlot. BP, biological process; CC, cellular component; MF, molecular function

To elucidate the specific functions and biological processes associated with differentially expressed genes, we conducted GO and KEGG pathway analyses. The GO enrichment analysis (Fig. 4) encompassed BP, CC, and MF domains. Among the downregulated genes, the enrichment scores primarily highlighted their involvement in biological processes, with the top three GO entries pertaining to the regulation of cellular processes (GO:0050794), activation of MAPK activity (GO:0000187), and the MAPK cascade (GO:0000165) in BP. In CC, they were associated with nucleoplasm (GO:0005654), cytoplasm (GO:0005737), and membrane-bounded organelles (GO:0043227). Molecular Function analyses revealed roles in protein binding activity (GO:0005515), S100 protein binding activity (GO:0044548), and growth factor activity (GO:0008083). Conversely, upregulated genes exhibited a smaller number of entries distributed nearly evenly, with the top entries across the three domains being the regulation of signal transduction (GO:0009966) in BP, the endomembrane system (GO:0012505) in CC, and RNA polymerase II cis-regulatory region sequence-specific DNA binding activity (GO:0000978) in MF.

**Fig. 5:**
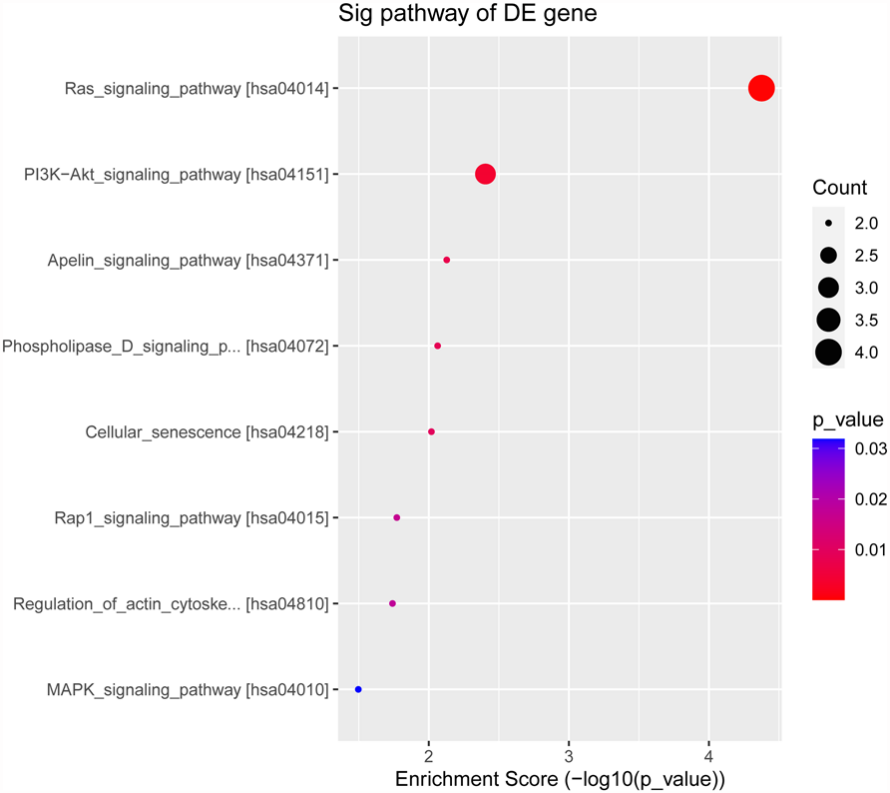
KEGG pathway analysis of significantly downregulated differentially expressed genes

Regarding KEGG pathway analysis, eight signaling pathways were identified among the significantly downregulated differentially expressed genes (Fig. 5). These pathways included the Ras signaling pathway (hsa04014), phosphatidylinositol-3-kinase (PI3K)–Akt signaling pathway (hsa04151), Apelin signaling pathway (hsa04371), phospholipase D signaling pathway (hsa04072), cellular senescence pathway (hsa04218), repressor/activator protein 1 signaling pathway (hsa04015), actin cytoskeleton regulatory pathway (hsa04810), and MAPK signaling pathway (hsa04010). However, no related signaling pathways were identified due to the limited number of downregulated differentially expressed genes.

### Constructing coexpression network

Differential expression analysis revealed that 283 lncRNA transcripts exhibited differential expression (*P* < 0.05, |log2FC| ≥ 0.585) between the POAF and Non-POAF groups, comprising 121 significantly upregulated and 162 significantly downregulated sequences. These transcripts were sorted in descending order based on |log2FC|. The top five transcripts were selected from both the upregulated and downregulated lncRNA collections. Subsequently, we cross-referenced the results of gene functional enrichment (GO and KEGG) to identify genes potentially associated with myocardial fibrosis and ion transport and confirmed their chromosomal locations. Among the remaining lncRNA transcript sequences, those with similar chromosomal locations to the aforementioned genes were selected.

**Fig. 6:**
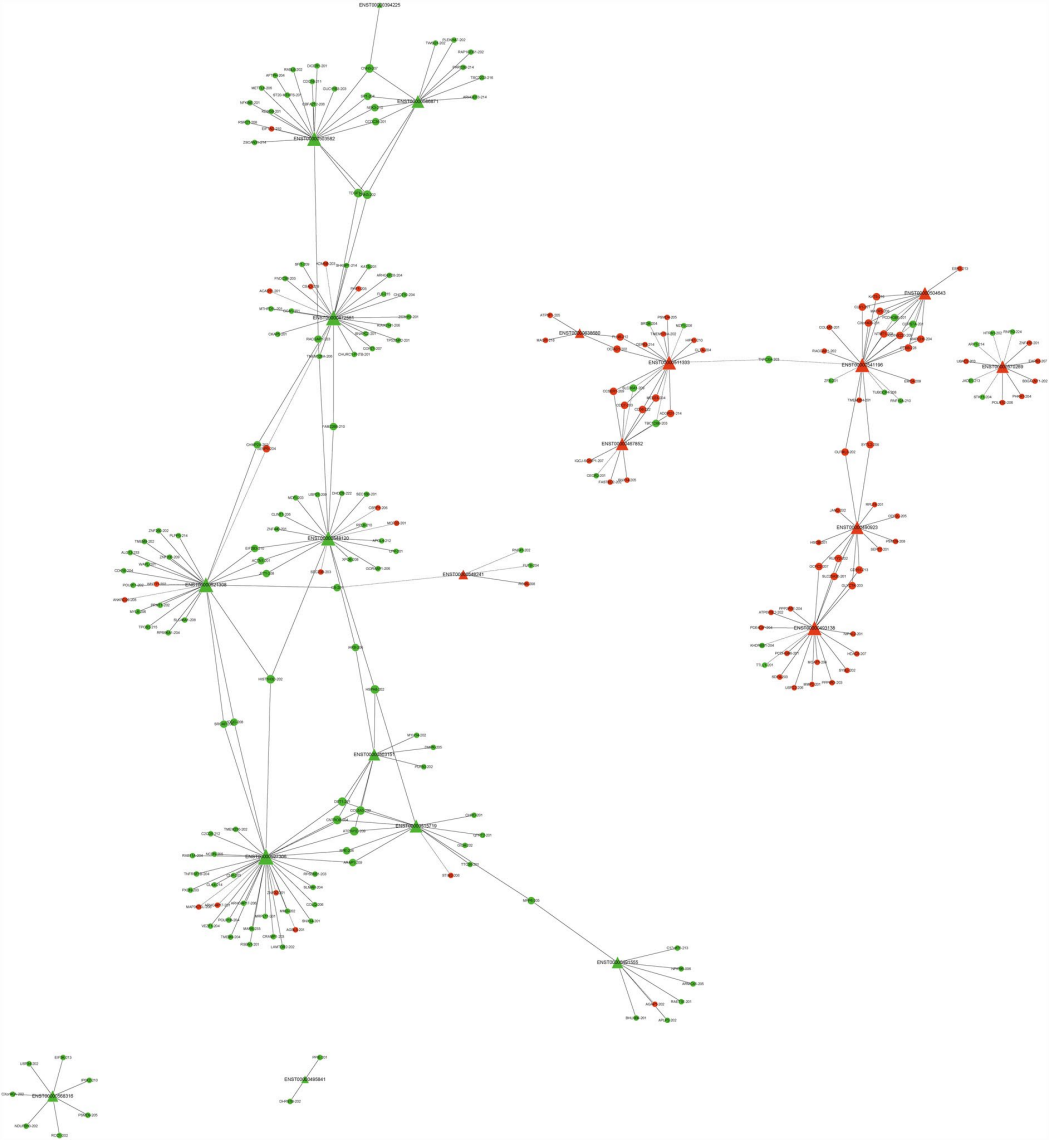
Analysis of lncRNA and mRNA coexpression network. The circle represents the coding gene, and the triangle represents lncRNAs. Red represents upregulation, and green represents downregulation. The solid line represents a positive correlation, and the dashed line represents a negative correlation

By employing these two selection methods, we identified a total of 12 downregulated and 9 upregulated lncRNA transcripts (detailed in Additional file 2: Table S2) for constructing a coexpression network. This network utilized significantly differentially expressed mRNA transcripts (*P* < 0.05, fold change > 1.5) to investigate the interactions between coding RNA and lncRNA (Fig. 6). This analysis involved 228 nodes (mRNA transcripts) connected to lncRNA transcripts. When sorted by correlation coefficient in descending order, the top five mRNAs were B3GALNT1-202, RPL18-201, CTSB-228, CXorf40B-201, and PPP6R3-203, with corresponding lncRNAs being ENST00000570269, ENST00000490923, ENST00000541196, and ENST00000493138, respectively.

## Discussion

The occurrence of atrial fibrillation following bypass surgery is influenced by factors such as cardiac-related surgery, cardiopulmonary bypass, and perioperative shock, with advanced age being a well-established risk factor. This study, by excluding extracorporeal circulation, meticulously examined the correlation between genetic molecules and POAF in patients undergoing off-pump CABG. The consistency in age, prevalence of diabetes, and hypertension between the Non-AF and POAF groups served to minimize confounding factors, ensuring the study's reliability.

Epicardial adipose tissue infiltration into myocardial tissue plays a pivotal role in promoting myocardial fibrosis through the secretion of adipo-fibrokines, significantly impacting cardiac function [15]. In this study, EAT was obtained from the epicardial surface adjacent to the right atrium and atrioventricular node. The unique anatomical structure of epicardial fat enables it to exert paracrine-like effects on adjacent myocardial tissue, influencing both its structure and function [20]. However, due to the limitations in sample size, the following discussion should be considered preliminary, with further research awaited.

Indeed, diabetic patients are at a higher risk of experiencing poorer outcomes after CABG surgery. We conducted separate subgroup analyses for diabetic and non-diabetic patient cohorts, but no significant differences were observed between these groups. Interestingly, our study [21] revealed a higher prevalence of SGLT2 inhibitor usage among diabetic patients in the Non-AF group. It is noteworthy that anti-diabetic medications, including SGLT2 inhibitors, known for their application in non-diabetic patients to enhance cardiovascular outcomes, may mitigate inflammatory distress and improve post-CABG results.

### mRNA function

Numerous significantly upregulated or downregulated coding genes in EAT are implicated in cardiovascular diseases, specifically myocardial remodeling and lipid metabolism, which are closely associated with atrial fibrillation occurrence.

For instance, PMEPA1 (ENSG00000124225.15_3, log_2_FC = – 1.36, *P* = 0.036) displayed a positive correlation with inflammation from tumor-infiltrating immune cells [22]. The delivery of the PMEPA1 gene can inhibit activin A activity, a molecule highly expressed in EAT that promotes myocardial/atrial fibrosis [15] in vitro. It can deactivate the activin/myostatin–SMAD3 pathway, thereby regulating muscle mass [23].

In our study, we identified that ENSG00000113578.17_2 [fibroblast growth factor 1 (FGF1)] and ENSG00000241186.9_3 [teratocarcinoma-derived growth factor-1 (TDGF1)] were enriched in multiple pathways associated with cell fibrosis, epithelial and endothelial cell migration (GO:0044344, GO:0071774, GO:0010595, GO:0010634, GO:000187, and GO:000165).

The KEGG results indicated that FGF1 played a crucial role in regulating the PI3K–Akt pathway (hsa04151), potentially inducing FGF1's neuroprotective effects against glutamate toxicity by inhibiting GSK-3β [24]. The interaction between GNG2 and MRAS inhibited Akt (a core component of the PI3K–Akt pathway) activity in an MRAS-dependent manner [25].

The PI3K–Akt pathway encompasses lipid kinases crucial for growth and metabolism, exerting a significant regulatory role in cardiac protection during stressful conditions [26]. This includes hepatic fibrosis [27], pulmonary fibrosis [28], and fibrosis within the cardiovascular system [29]. The PI3K–Akt signaling pathway has been implicated in the regulation of cardiac fibrosis occurrence and pathological progression [30] by governing cell survival, apoptosis, growth, and cardiac contractility, ultimately leading to heart failure [31]. Inhibition of cardiac PI3K–Akt signaling has been linked to an increased risk of atrial fibrillation [32]. Mice with reduced PI3K activation exhibited compromised cardiac function, more pronounced atrial enlargement, and fibrosis, thereby significantly increasing susceptibility to atrial fibrillation [33]. PI3K plays a pivotal cardioprotective role [34], and its increased activity can extend mouse lifespan by 15% to 20% [26].

The POAF group displayed lower total triglyceride and total cholesterol levels compared to the Non-POAF group. This suggests that while lower blood lipid levels are protective against coronary heart disease, they may be associated with an increased risk of POAF—a seemingly paradoxical phenomenon. A prior retrospective clinical study conducted in our center and several clinical investigations [35–37] have reported a negative correlation between preoperative LDL levels and POAF occurrence. However, the underlying mechanism behind this association remains unclear.

FGF1 is significantly downregulated and closely linked to lipid metabolism [38]. An upsurge in FGF1 levels corresponds to increases in TC, TG, and LDL levels [39], and there exists a positive correlation between FGF1 mRNA expression and cholesterol levels [40]. In our study, the lower blood lipid levels observed in the POAF group may be closely related to the downregulated FGF1 mRNA.

### lncRNA transcripts

LncRNAs, which exceed 200 nucleotides in length [41], typically function as transcriptional or posttranscriptional regulators involved in chromatin remodeling and splicing. They are associated with various pathological processes [41].

We established a coding–noncoding gene coexpression (CNC) network to investigate the potential role of differentially expressed lncRNA transcripts in the POAF group. The top 10 mRNA transcripts with the highest upregulated and downregulated differential expression ratios displayed significant correlations with lncRNAs within the CNC network, and these mRNA transcripts were under the regulatory influence of lncRNA transcripts.

Several differentially expressed mRNA transcripts were concurrently regulated by multiple lncRNA transcripts. For instance, COL6A3-209 exhibited downregulation in tandem with the decreased expression of lncRNAs ENST00000527306 (HOOK3-204) and ENST00000515719 (CLPTM1L-215). COL6A3 is known to promote the formation of collagen fibers, consequently inducing myocardial fibrosis and expediting atrial remodeling [42]. Furthermore, mRNA transcripts such as IARS-206 [43], TDGF1 [44], and CNN3-202 [45], regulated by two or more lncRNAs, demonstrated close associations with myocardial or hepatic fibrosis.

SRCAP, which experienced downregulation alongside the decreased expression of lncRNAs ENST00000527306 (HOOK3-204) and ENST00000521308 (CCDC69-205), has the potential to influence the levels of sarcoplasmic reticulum Ca^2+^ regulatory proteins and cardiac Ca^2+^ homeostasis. The loss of its core subunit Znhit1 has been linked to postnatal arrhythmias and rapid heart failure in mice [46]. Notably, ENST00000521308 (CCDC69-205) exhibited the most significant downregulation (log_2_FC = – 2.60, *P* = 0.002) among all significantly differentially expressed lncRNA transcripts.

## Limitations

The limited sample size in this study may affect the reliability of the results. Larger studies are necessary to validate these findings. Although we have explored the potential relationship between lncRNAs and coding RNAs, further experimental and clinical verifications are essential to establish a causal link and elucidate specific action pathways.

## Conclusions

This study represents the first examination of the RNA expression profile of EAT in patients with nonvalvular POAF. Gene function and coexpression network analyses have unveiled the likelihood of co-regulation between mRNAs and lncRNAs in myocardial fibrosis or ion channel regulation, which may be closely associated with the onset of POAF. This genetic perspective has identified potential targets for POAF, and the discovery of key lncRNAs may pave the way for further clinical and fundamental research.

### Supplementary Information

**Additional file 1: Table S1.** Primer lists were used for Quantitative real-time polymerase chain reaction.

**Additional file 2: Table S2.** Results of differential expression analysis of LncRNA selected for co-expression analysis.

**Additional file 3: Table S3.** Demographic and clinical characteristics of patient in subgroup.

**Additional file 4: Table S4.** Medication on admission and medication on discharge for the patient cohort.

## Data Availability

The datasets generated and analyzed in this study can be accessed in the Gene Expression Omnibus (GEO) under the accession number [GSE222739, https://www.ncbi.nlm.nih.gov/geo/query/acc.cgi?acc=GSE222739]."

## Abbreviations

POAF: Postoperative atrial fibrillation
CABG: Coronary artery bypass grafting
BMI: Body mass index
BNP: Brain Natriuretic Peptide
GLU: Glucose
HbA1C: Glycosylated Hemoglobin
TC: Total Cholesterol
HDL: High Density Lipoprotein
LDL: Low Density Lipoprotein
TG: Triglyceride
Lpa: Apolipoprotein A
BNU: Blood Urea Nitrogen
Scr: Serum creatinine
LVEDD: Left ventricular end-diastolic dimension
LVEF: Left ventricular ejection fraction
LAD: Left atrial diameter
RAD: Right atrial diameter
ICU: Intensive care unit
