# Supplementary material for: Long noncoding RNA and messenger RNA profiling in epicardial adipose tissue of patients with new-onset postoperative atrial fibrillation after coronary artery bypass grafting
Source: Eur J Med Res. 2024 Feb 17;29:134. doi: 10.1186/s40001-024-01721-x (PMC10874008; doi:10.1186/s40001-024-01721-x)
Supplement: Supplementary file 1 — Additional file 1: Table S1. Primer lists were used for Quantitative real-time polymerase chain reaction. [file 40001_2024_1721_MOESM1_ESM.doc]

Table S1. Primer lists were used for Quantitative real-time polymerase chain reaction

| gene (LncRNA) | Bidirectional primer sequence | Length of Product(bp) |
| --- | --- | --- |
| β-actin（H） | F:5' GTGGCCGAGGACTTTGATTG3'  R:5’ CCTGTAACAACGCATCTCATATT3’ | 73 |
| ENST00000490923 | F:5' GTGCTAACTTGCCCTCTGCC 3’  R:5’ CTCGGCCATACCAATCTGCC 3’ | 160 |
| ENST00000491555 | F:5' CATCTCCCTCTACCAGAATGAC 3’  R:5’ TGCCGGTATTTCCAGTCAGC 3’ | 89 |
| ENST00000504643 | F:5' GTCGATGCCAGCTTCTTCTTG 3’  R:5’ TTGTCCATACAGTACCACTATGT 3’ | 155 |
| ENST00000515719 | F:5' CTTTCTGCAGGGGTCTAT 3’  R:5’ TTCACTCTGCGTTTATCCAC 3’ | 257 |
| ENST00000521308 | F:5' CCCCGAAAAAGCTATAACTGTCC 3’  R:5’ AGGACCCTTTGCTGCTCATC 3’ | 177 |
| ENST00000527306 | F:5' AGGATCAAGGCTCAAAAGCAGA 3’  R:5’ TCAACATTGTGAAACTGATGGACC 3’ | 75 |

The F means forward primer; the R means reversed primer.
