# Supplementary material for: Long noncoding RNA and messenger RNA profiling in epicardial adipose tissue of patients with new-onset postoperative atrial fibrillation after coronary artery bypass grafting
Source: Eur J Med Res. 2024 Feb 17;29:134. doi: 10.1186/s40001-024-01721-x (PMC10874008; doi:10.1186/s40001-024-01721-x)
Supplement: Supplementary file 2 — Additional file 2: Table S2. Results of differential expression analysis of LncRNA selected for co-expression analysis. [file 40001_2024_1721_MOESM2_ESM.docx]

Table S2. Results of differential expression analysis of LncRNA selected for co-expression analysis

|  | Track_id | Gene_Name | Locus | Strand | Trans_Type | log2FC | Fold_Change | p_value |
| --- | --- | --- | --- | --- | --- | --- | --- | --- |
| down-regulated (12) | ENST00000521308.5_1 | CCDC69-205 | chr5:150560613-150603654 | - | processed_transcript | -2.60054 | 0.164876719 | 0.002271 |
|  | ENST00000568316.1_1 | RBBP6-211 | chr16:24550906-24551605 | + | retained_intron | -2.16616 | 0.222802701 | 0.049423 |
|  | ENST00000527306.1_1 | HOOK3-204 | chr8:42752089-42853299 | + | retained_intron | -1.96966 | 0.255313023 | 0.002528 |
|  | ENST00000394225.6_1 | NDUFC1-202 | chr4:140211107-140223680 | - | processed_transcript | -1.87427 | 0.272764171 | 0.047286 |
|  | ENST00000515719.5_1 | CLPTM1L-215 | chr5:1318414-1321944 | - | retained_intron | -1.77299 | 0.292601351 | 0.021207 |
|  | ENST00000503151.5_1 | CLPTM1L-203 | chr5:1322949-1335236 | - | retained_intron | -1.16584 | 0.445705049 | 0.020094 |
|  | ENST00000491555.5_1 | IPO5-223 | chr13:98653900-98674757 | + | retained_intron | -0.99594 | 0.501408724 | 0.043128 |
|  | ENST00000586871.5_1 | AC020907.2-201 | chr19:35629728-35634266 | + | processed_transcript | -0.93189 | 0.524170638 | 0.045988 |
|  | ENST00000503582.1_1 | SPRY4-203 | chr5:141694674-141703982 | - | processed_transcript | -0.90624 | 0.533574869 | 0.03719 |
|  | ENST00000472561.5_1 | RASSF4-208 | chr10:45467242-45488878 | + | processed_transcript | -0.66687 | 0.629871802 | 4.93E-07 |
|  | ENST00000548120.1_1 | RNF41-204 | chr12:56600371-56602328 | - | retained_intron | -0.61677 | 0.652130282 | 0.00352 |
|  | ENST00000495841.1_1 | CAV2-214 | chr7:116140098-116146407 | + | processed_transcript | -0.61081 | 0.654830863 | 0.048405 |

|  | Track_id | Gene_Name | Locus | Strand | Trans_Type | log2FC | Fold_Change | p_value |
| --- | --- | --- | --- | --- | --- | --- | --- | --- |
| up-regulated (9) | ENST00000570269.2_3 | AC093010.3-201 | chr3:114033348-114048561 | - | 3prime_overlapping_ncRNA | 2.556167 | 5.881431 | 0.032853 |
|  | ENST00000467852.1_1 | VPS26A-204 | chr10:70884002-70928384 | + | processed_transcript | 2.513019 | 5.708134 | 0.04002 |
|  | ENST00000541196.2_1 | HCP5-204 | chr6:31430959-31433481 | + | sense_overlapping | 2.269875 | 4.822812 | 0.041099 |
|  | ENST00000638680.1_1 | SCARB2-213 | chr4:77080067-77135029 | - | retained_intron | 2.153805 | 4.45 | 0.025197 |
|  | ENST00000490923.5_1 | CSAD-217 | chr12:53553671-53564496 | - | retained_intron | 1.56567 | 2.960149 | 0.036973 |
|  | ENST00000504643.5_1 | MATR3-210 | chr5:138629672-138658637 | + | retained_intron | 1.133814 | 2.194381 | 0.018656 |
|  | ENST00000511333.5_1 | MATR3-217 | chr5:138651036-138652794 | + | retained_intron | 0.849393 | 1.801743 | 0.00627 |
|  | ENST00000548241.1_1 | ANKRD52-203 | chr12:56631591-56638511 | - | processed_transcript | 0.831439 | 1.77946 | 0.037384 |
|  | ENST00000493138.5_1 | GUK1-235 | chr1:228328008-228336578 | + | processed_transcript | 0.760359 | 1.693912 | 0.014374 |
