# Supplementary material for: Long noncoding RNA and messenger RNA profiling in epicardial adipose tissue of patients with new-onset postoperative atrial fibrillation after coronary artery bypass grafting
Source: Eur J Med Res. 2024 Feb 17;29:134. doi: 10.1186/s40001-024-01721-x (PMC10874008; doi:10.1186/s40001-024-01721-x)
Supplement: Supplementary file 3 — Additional file 3: Table S3. Demographic and clinical characteristics of patient in subgroup. [file 40001_2024_1721_MOESM3_ESM.docx]

Table S3. Demographic and clinical characteristics of patient in subgroup

| Characteristics | diabetic(n=5) | non-diabetic (n=5) | P value |
| --- | --- | --- | --- |
| POAF,(n %) | 2 (40%) | 3 (60%) | 0.580 |
| Leukocytes, ×10^9^/L | 6.4±1.6 | 5.8±0.9 | 0.518 |
| Neutrophils | 3.8±1.1 | 3.3±0.4 | 0.350 |
| Lymphocytes | 2.0±0.5 | 1.8±0.6 | 0.565 |
| Neutrophil ratio | 56.1 (54.4-62.1) | 56.1 (53.8-58.2) | 0.548 |
| Lymphocytes ratio | 27.9 (26.0-32.9) | 34.1 (32.8-35.9) | 0.690 |
| Hemoglobin, g/L | 137.2±22.1 | 134.6±23.7 | 0.862 |
| BNP, ng/L | 85.6±50.2 | 97.2±82.3 | 0.796 |
| GLU, | 7.2±2.5 | 6.3±3.6 | 0.428 |
| HbA1C | 8.2±2.0 | 5.9±0.2 | 0.035 |
| TC, mmol/L | 4.0±1.1 | 3.4±1.4 | 0.474 |
| LDL, mmol/L | 2.5±0.9 | 1.7±0.9 | 0.173 |
| HDL, mmol/L | 0.8±0.1 | 0.8±0.2 | 0.882 |
| TG, mmol/L | 2.0±0.9 | 1.3±0.9 | 0.294 |
| Lpa, mmol/L | 21.2±13.0 | 13.0±4.0 | 0.219 |
| BUN, mmol/L | 6.4±1.6 | 6.0±1.6 | 0.684 |
| Scr, μmol/L | 63.9±18.1 | 74.0±17.3 | 0.395 |
| LVEDD, mm | 48.4±3.0 | 47.4±3.4 | 0.635 |
| LVEF, % | 59.6±5.4 | 56.8±11.4 | 0.634 |
| LAD, mm | 39.8±1.9 | 41.9±3.7 | 0.286 |
| RAD, mm | 38.4±1.6 | 40.7±3.4 | 0.207 |
| Anastomoses | 2.4±1.3 | 2.4±1.3 | 1.000 |
| Drainage-1 | 378.0±95.0 | 372±59.0 | 0.907 |
| Drainage-T | 795.0±202.1 | 1140.0±539.0 | 0.217 |
| Length of ICU stay | 4.8±1.8 | 5.2±1.9 | 0.742 |

BMI, Body mass index; Drainage-1, BNP, Brain Natriuretic Peptide; HbA1C, Glycosylated Hemoglobin; TC, Total Cholesterol; LDL, Low Density Lipoprotein; HDL, High Density Lipoprotein; TG, Triglyceride; Lpa, Lipoprotein A; BUN, Blood Urea Nitrogen; Scr, Serum creatinine; LVEDD, left ventricular end-diastolic dimension; LVEF, left ventricular ejection fraction; LAD, Left atrial diameter; RAD, Right atrial diameter; Drainage-1, Drainage on the first postoperative day; Drainage-T: Total postoperative drainage; ICU, Intensive care unit.
