# Supplementary material for: Long noncoding RNA and messenger RNA profiling in epicardial adipose tissue of patients with new-onset postoperative atrial fibrillation after coronary artery bypass grafting
Source: Eur J Med Res. 2024 Feb 17;29:134. doi: 10.1186/s40001-024-01721-x (PMC10874008; doi:10.1186/s40001-024-01721-x)
Supplement: Supplementary file 4 — Additional file 4: Table S4. Medication on admission and medication on discharge for the patient cohort. [file 40001_2024_1721_MOESM4_ESM.docx]

| **Clinical characteristics** | | **POAF** | **Non-AF** | **P-value** |
| --- | --- | --- | --- | --- |
| **Total n=10** | | **n=5** | **n=5** |  |
| Medication  on  admission | ACEI/ARB[n(%)] | 2(40) | 2(40) | 1.000 |
|  | βRB[n(%)] | 4(80) | 4(80) | 1.000 |
|  | CCB[n(%)] | 2(40) | 2(40) | 1.000 |
|  | Diuretics[n(%)] | 0(0) | 0(0) | 1.000 |
|  | Nitrate[n(%)] | 5(100) | 5(100) | 1.000 |
|  | Aspirin[n(%)] | 3(60) | 3(60) | 1.000 |
|  | Statins[n(%)] | 5(100) | 4(80) | 0.292 |
|  | Metformin[n(%)] | 2(40) | 1(20) | 0.490 |
|  | Acarbose[n(%)] | 2(40) | 0(0) | 0.114 |
|  | SGLT2I[n(%)] | 0(0) | 2(40) | 0.114 |
|  |  |  |  |  |
| Medication  on  discharge | ACEI/ARB[n(%)] | 3(60) | 4(80) | 0.490 |
|  | βRB[n(%)] | 5(100) | 5(100) | 1.000 |
|  | CCB[n(%)] | 4(80) | 4(80) | 1.000 |
|  | Diuretics[n(%)] | 5(100) | 5(100) | 1.000 |
|  | Nitrate drugs[n(%)] | 5(100) | 5(100) | 1.000 |
|  | Aspirin[n(%)] | 5(100) | 5(100) | 1.000 |
|  | Clopidogrel[n(%)] | 5(100) | 5(100) | 1.000 |
|  | Statins[n(%)] | 5(100) | 5(100) | 1.000 |
|  |  |  |  |  |

**Table S4.** Medication on admission and medication on discharge for the patient cohort
